# Imaging findings of gastrointestinal tract tumors in children and adolescents

**DOI:** 10.1186/s13244-022-01193-9

**Published:** 2022-03-22

**Authors:** H. Nursun Özcan, Özlem Özkale Yavuz, Saniye Ekinci, Berna Oguz, Tezer Kutluk, Mithat Haliloglu

**Affiliations:** 1 Department of Radiology/Subdivision of Pediatric Radiology, Hacettepe University School of Medicine Ankara, Sıhhiye, 06100 Ankara Turkey; 2 Department of Pediatric Surgery, Hacettepe University School of Medicine Ankara, Sıhhiye, Turkey; 3 Division of Pediatric Oncology, Department of Pediatrics, Hacettepe University School of Medicine Ankara, Sıhhiye, Turkey

**Keywords:** Pediatric, Mass, Colon, CT, MRI

## Abstract

Gastrointestinal (GI) tract tumors are rarely seen in children and adolescents, and can easily be misdiagnosed. Lymphoma is the most frequent GI tract tumor, and the common locations are ileum and ileocecal area. GI tract tumors may present as large heterogeneous mass lesions. For gastric and colonic tumors, increased wall thickening usually prompts the diagnosis of GI tract tumors. Computed tomography and magnetic resonance imaging might be used in clinically suspected cases for correct/appropriate diagnosis and management. Awareness as regards the most common tumors and their locations is paramount for radiologists. Likewise, the aim of this article was to define the imaging findings of primary benign and malignant GI tract tumors in children and adolescents.

## Key points

- GI tract tumors are rarely seen in children and adolescents and can easily be misdiagnosed.
- For the diagnosis of GI tract tumors, increased wall thickening is usually suggestive.
- In children, ultrasound is the first imaging method to evaluate abdominal pathology. Most GI tract tumors in children are diagnosed by US after which CT and MRI should follow in clinically suspected cases.

## Introduction

Cancer arising from the gastrointestinal (GI) tract is uncommon in the pediatric age group. No more than 5% of childhood tumors are seen in the GI tract [1]. Due to their rare existence, the current literature contains only review of small case series and anecdotal reports which have been mainly published in surgery journals. The most common GI tract tumors are gastrointestinal lymphoma, colorectal carcinoma, gastrointestinal stromal tumors (GIST), inflammatory pseudotumor and gastric tumors (signet ring cell carcinoma, adenocarcinoma, etc.) [2–7]. The purpose of this paper was to review the most commonly seen primary benign and malignant GI tract tumors of the esophagus, stomach, small intestine and colon (Table 1) as well as their imaging features using ultrasonography (US), computed tomography (CT), and magnetic resonance imaging (MRI).

**Table 1:** The most frequent types of gastrointestinal tract tumors

Esophagus	Stomach	Small bowel	Large bowel
Adenoma	Polyp	Adenoma	Polyp
Leiomyoma	Lymphoma	Hamartoma	Lipoma
Adenocarcinoma	GIST	Lipoma	Leiomyoma
Squamous cell carcinoma	Leiomyoma/Leiomyosarcoma	Leiomyoma	Neurofibroma
Lymphoma	Teratoma	Lymphoma	Lymphoma
Leiomyosarcoma	Inflammatory pseudotumor	GIST	Adenocarcinoma
		Neuroendocrine tumors	Neuroendocrine tumors
		Adenocarcinoma	
		Inflammatory pseudotumor	

## Colon and small intestine

### Colorectal carcinoma

**Fig. 1:**
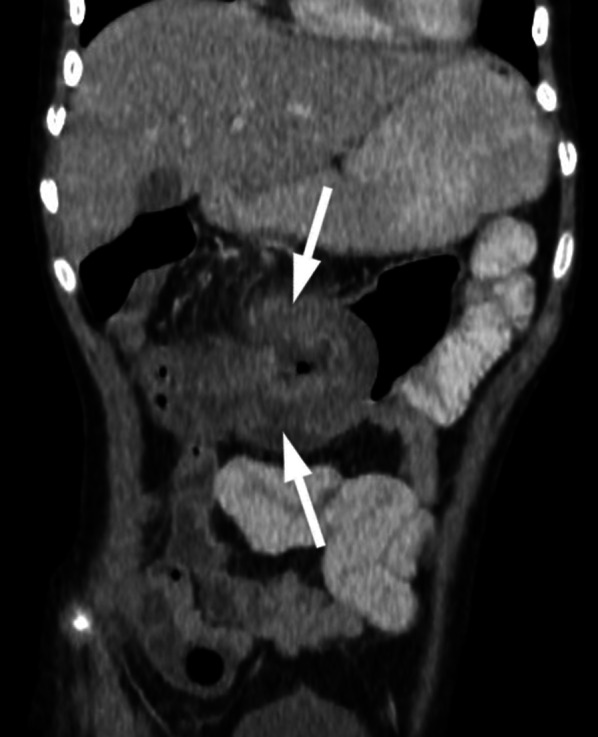
An 11-year-old girl was admitted with abdominal pain in the last two days. On physical examination, she had abdominal tenderness. On US, there was a suspicious appearance of intussusception. Contrast-enhanced coronal reformatted CT image shows increased wall thickening at the transverse colon seen as intussusception (arrows) and increased mesenteric density. Segmental colon resection was performed, and the histopathological diagnosis was colon adenocarcinoma

Colorectal carcinoma is extremely uncommon in the pediatric age group. Most common complaints are abdominal pain and vomiting [4–6]. Less common symptoms are abdominal distension, palpable abdominal mass, or alteration in bowel habits. Signs and symptoms of the colorectal carcinoma in the pediatric age group are nonspecific and can mimic those of acute abdomen—including intussusception, intestinal obstruction and appendicitis (Fig. 1).

Regarding colon adenocarcinoma, 10% of the patients have underlying causes like familial adenomatous polyposis, other polyposis syndromes, Lynch syndrome, and ulcerative colitis [4]. 50% of pediatric colon carcinomas are mucinous adenocarcinomas, in contrast to adults who frequently have adenocarcinoma. A propensity for sigmoid and transverse colon disease is present in adolescent patients. In children and adolescents, the prognosis of colorectal carcinoma is worse than in adults. The poor prognosis is usually related to diagnostic delays. Most of the children and adolescents are diagnosed with mucinous and/or signet ring cell variants of the colorectal carcinoma which have poor prognosis. Advanced level of the tumor at the time of initial diagnosis is due to the diagnostic delay and majority of the children have metastasis to local lymph nodes and liver, and in some cases they have peritoneal carcinomatosis. Clinical suspicion for this tumor is crucial to prevent delay in the diagnosis of pediatric patients.

**Fig. 2:**
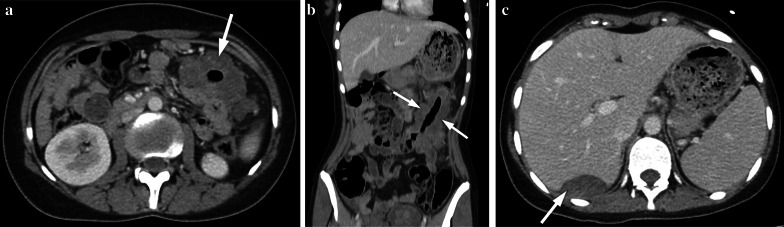
**a–c** A 16-year-old girl presented with complaints of fatigue, abdominal pain and diarrhea for the last 6 months. CT examination was performed when a suspicious mass was seen in the liver on US. **a–c** Contrast-enhanced axial and coronal reformatted CT images show diffuse increased wall thickening at the splenic flexure and transverse colon (arrows). Note the subcapsular metastatic lesion adjacent to the right liver lobe (arrow). Ca-125 level was found to be high (234 U/mL, normal range 0–35 U/mL). The patient was operated and the histopathological diagnosis was colon mucinous adenocarcinoma

**Fig. 3:**
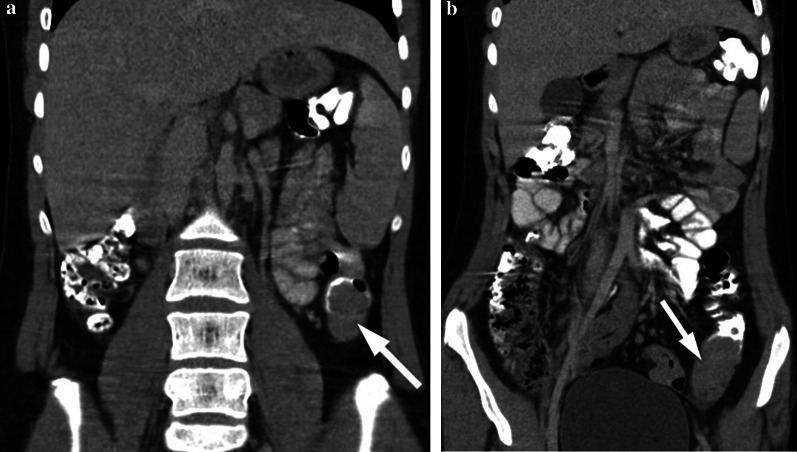
**a, b** A 17-year-old girl with rectal bleeding for the last 2 weeks. Hb level was 9.8 gr/dL (normal range 11.7–15.5 gr/dL). Colonoscopy (performed in an external center) showed many irregular mucosal polyps in the descending/sigmoid colon and rectum. Biopsy revealed villous adenocarcinoma. The patient underwent total colectomy. **a, b** Contrast-enhanced coronal reformatted CT images show hypodense polypoid tumoral lesions in the descending colon (arrows)

Similar to adults, diagnosis of adenocarcinoma in children necessitate colonoscopy and imaging studies (US, CT/MRI). Barium studies are not used nowadays. US can be helpful while imaging colonic wall and perimesenteric fat tissues. In US imaging, the colon wall is normally 2–3 mm thick and has 5–8 layers. Carcinomas are usually hypoechogenic and can be seen as bowel wall thickening with disrupted integrity or sometimes as lobulated masses. Lymph nodes involved by the tumor are round and they lose the fatty hilum. CT and MRI are necessary for local/distal extension of the tumor to the peritoneal cavity, retroperitoneal lymph nodes and/or metastasis. Colorectal carcinomas can appear as focal, irregular wall thickening, pericolonic fat tissue infiltration, serosal irregularity and with lymphadenopathy on CT (Figs. 2, 3). Calcification can be seen in mucinous carcinoma. In the early stage of small tumors without wall thickening, arterial contrast enhancement might be the only imaging finding. Herewith, the presence of thickening and invasion of the wall layers is remarkable when evaluating advanced tumor stages. The risk of lymph node metastasis has increased in younger patients (< 40 years) with early-stage colon adenocarcinomas [7, 8]. In histopathological examinations, it has been depicted that nearly 50% of the lymph nodes in colon cancer are smaller than 5 mm in terms of size [8]. Lymph node size > 1 cm, short-long axis diameter ratio, internal heterogeneity, irregular outer border, attenuation values > 100 Hounsfield units (HU), and cluster of three or more normal sized lymph nodes, or any combination of the above, have all been used as a combined criteria [8]. Low-density lymph nodes can also be present due to the predominantly extracellular mucin component of the tumor in mucinous adenocarcinoma [9].

**Fig. 4:**
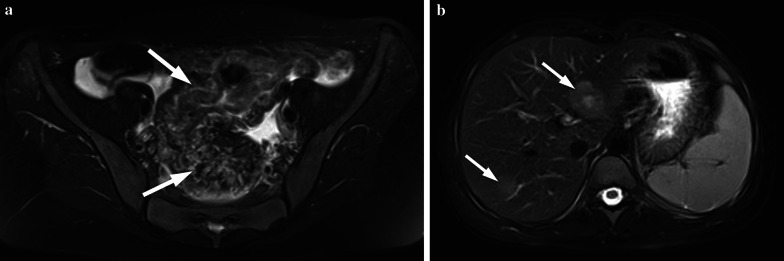
**a, b** A 14-year-old girl with colon adenocarcinoma. She had blood in her stool and tenesmus for the last 1 month. Hb level was 6.3 gr/dL (normal range 11.7–15.5 gr/dL), Ca 19–9: 539 U/mL (normal range 0–35 U/mL), CeA: 207 ng/mL (normal range 0–7 ng/mL). US showed multiple lesions in the liver with hypoechoic halo. **a** Axial fat-saturated T2-weighted MRI demonstrates increased wall thickening at the sigmoid and rectal colon (arrows). **b** Axial fat-saturated T2-weighted MRI shows mild hyperintense liver metastases (arrows). She was operated

**Fig. 5:**
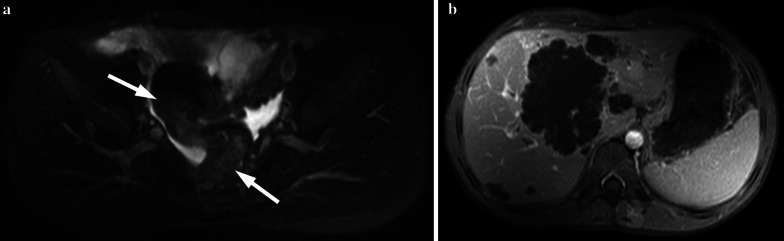
**a, b** A 14-year-old boy with colon mucinous adenocarcinoma. He had back pain and loss of appetite for the last 1.5 months. His father and uncle had colon carcinoma. **a** Axial fat-saturated T2-weighted image demonstrates diffuse increased wall thickening at the sigmoid colon (arrows). **b** Post-contrast T1-weighted image shows hypoenhancing liver metastases

MRI is another imaging method to detect the transmural tumor infiltration and to screen for lymph node involvement. The majority of colorectal carcinomas have soft tissue intensity that narrows the bowel lumen—easily seen in all imaging techniques. Colorectal carcinomas are generally demonstrated as hypointense masses on T1-weighted images and hyperintense-heterogeneous masses on T2-weighted images (Fig. 4). Invasion of the bowel wall layers with the tumor, after the infiltration of peripheral adipose tissue, is well observed on T1-weighted sequences. Penetration and invasion of bowel wall layers are best demonstrated on T2-weighted sequences (Fig. 5). Disappearance of hyperintense adipose tissue between the bowel wall and neighboring organs or other intestinal loops is considered as extracolonic local and regional extension [10]. On diffusion-weighted images, restricted diffusion is observed owing to the tumoral cellularity, not only in the tumor but also, in the metastases. Both CT and MRI can assess the peritoneal carcinomatosis in the presence of ascites, peritoneal nodules, mesenteric thickening and fat stranding all of which signify peritoneal spread of the disease. Inflammatory bowel disease and bowel lymphoma should be kept in mind as regards the differential diagnosis. The management of colorectal cancer in children is similar to adults. Complete surgical excision is the most important prognostic factor. Surgery/complete tumor resection involving the lymphatic pathway of the affected colon segment is the main treatment for carcinoma in adolescent patients. Adjuvant chemotherapy and radiotherapy are the other options in the treatment of colorectal carcinoma with distant extension.

### Inflammatory myofibroblastic tumor

**Fig. 6:**
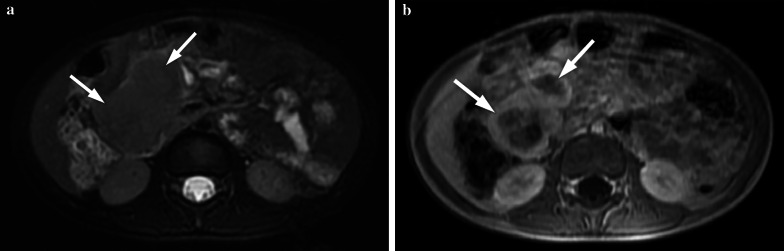
**a, b** A 3-year-old girl with inflammatory pseudotumor. She had fever and weight loss for the last 3–4 months. In physical examination, a mass was detected in the abdomen. **a** Axial fat-saturated T2-weighted image shows hypointense tumor in the ileum (arrows). **b** Post-contrast T1-weighted axial image shows peripherally contrast-enhanced tumor with central necrotic parts (arrows). After biopsy, ALK and SMA positive inflammatory pseudotumor was diagnosed

Inflammatory myofibroblastic tumor (IMT) or inflammatory pseudotumor is used to define a benign and rare process particularly involving the lung and orbit, also seen at almost every site in the body. Pseudotumors of the GI system are uncommon, and they mainly involve the stomach, followed by small and large intestines. It may be asymptomatic or manifest with abdominal pain, intestinal obstruction, dysphagia, anemia, and fever. The radiologic features are non-specific and include wall infiltration and extraluminal extension [11]. On US, they appear like hypoechoic or hyperechoic solid masses with variable vascularity on Doppler imaging. CT shows well-defined, hypodense, heterogeneous masses in non-enhanced images. After injection of the contrast material, IMTs are seen as homogeneous or heterogeneous lesions with variable enhancement on delayed acquisitions (due to the presence of fibrous tissue formed by the accumulation of myofibroblasts and fibroblast). MR images display hypointense masses on both T1- and T2-weighted images, representing the fibrotic tissue (Fig. 6). Their characteristic post-contrast enhancement pattern is hypointense lesion in the arterial phase, with progressive-variable enhancement in the delayed phase. Abdominal IMT should be considered in the differential diagnosis of any soft-tissue mass within in the abdomen. Despite all the imaging findings, histopathological confirmation is needed for accurate diagnosis.

The most common treatment is surgery, although local recurrence is higher in the GI tract when compared with other locations of involvement. Steroids, NSAIDs and radiotherapy are the other adjunctive treatments [12].

### Lymphoma

Non-Hodgkin’s lymphoma (NHL) is the most frequent tumor of the GI tract in children, and it is frequently of B cell origin. Burkitt lymphoma is the most frequent subtype of non-Hodgkin's lymphoma in childhood. Concerning extranodal involvement, GI tract involvement has been reported in 22.5% of cases, with abdominal or pelvic masses in 45% of the cases [13]. Distal ileum, cecum, appendix, and ascending colon are the most common sites of involvement; however, the stomach and duodenum are very rarely involved. The presentation varies from no symptoms (with an obscure abdominal mass) to more urgent clinical scenarios like intestinal obstruction with bilious emesis or abdominal pain due to perforation. Of note, intussusception can be another presentation in children [14].

**Fig. 7:**
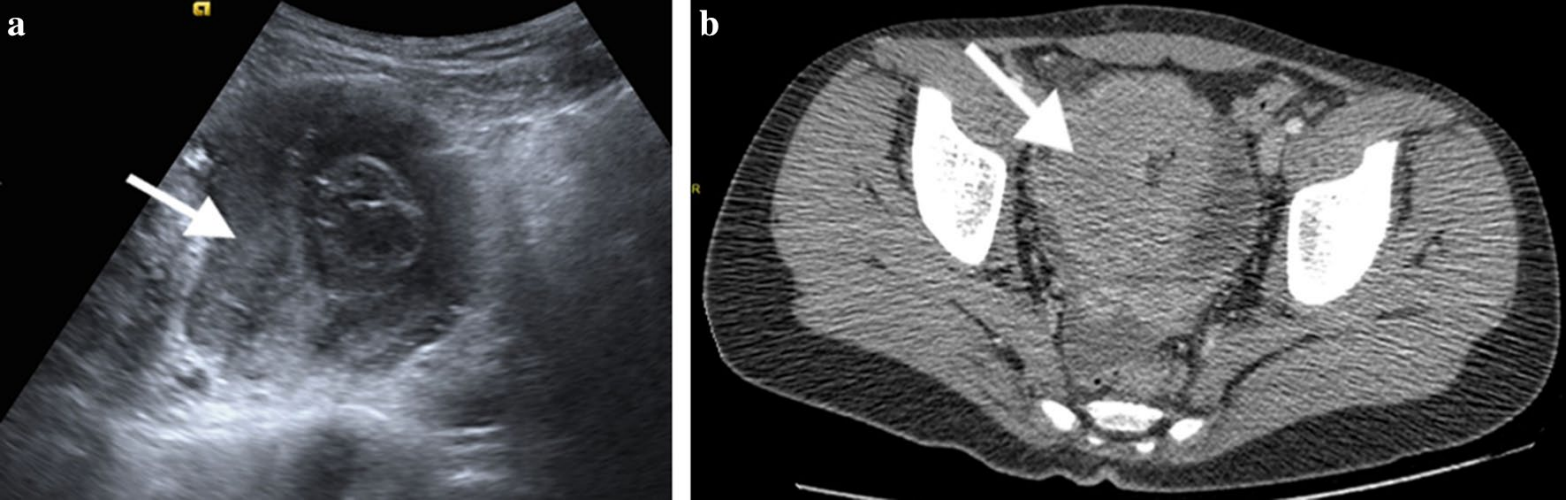
**a, b** A 11-year-old girl with Burkitt lymphoma. She complained of abdominal pain for the last 2 months. **a** US image demonstrates pathological diffuse thickening of the intestinal wall (arrow) causing intussusception at the level of the ileal loops and appearance of a pseudo-mass. **b** Axial post-contrast CT image shows mass lesion (arrow) in the right lower quadrant in the intestinal wall. Also note the pelvic fluid

**Fig. 8:**
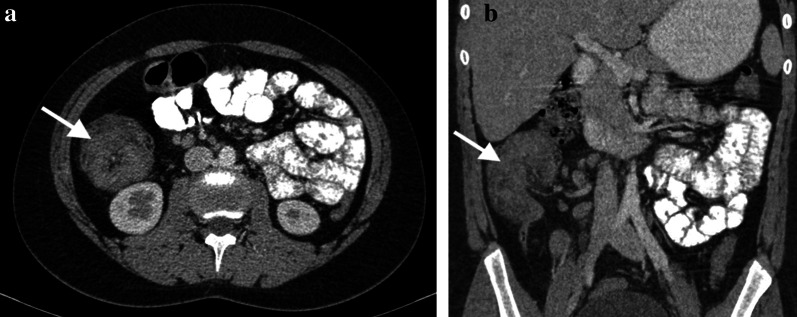
**a, b** A 13-year-old boy with Burkitt lymphoma. The patient had abdominal pain and vomiting for the last 15 days. Invagination was detected in US performed in an external center. Axial and coronal reformatted CT images show intussusception at the level of the ileocecal valve (arrows)

**Fig. 9:**
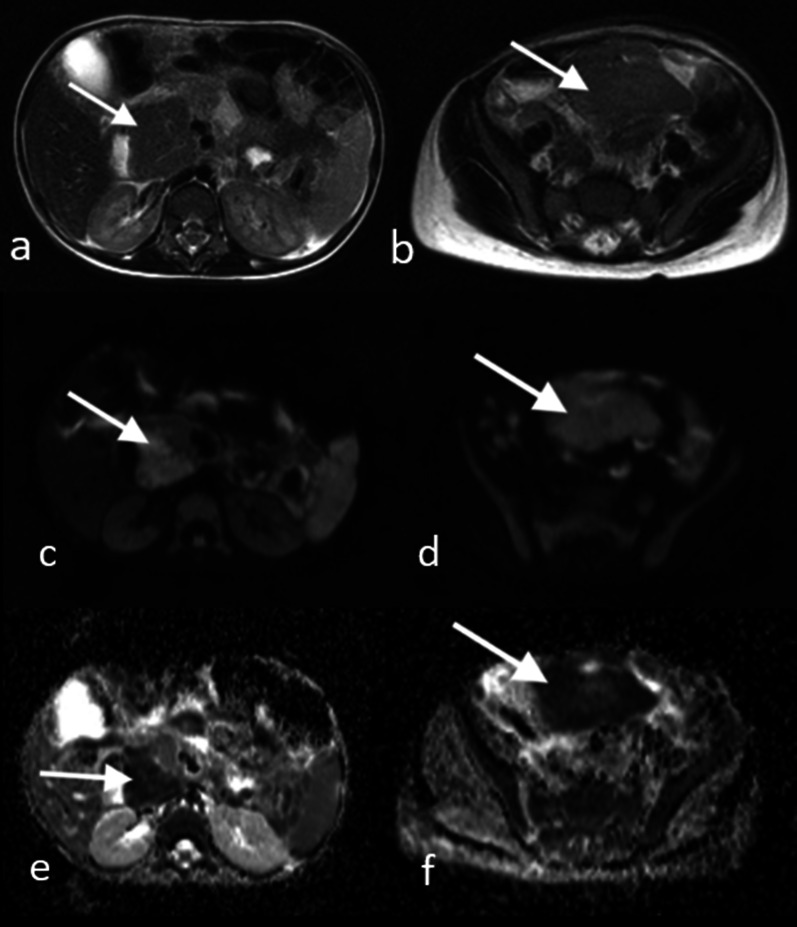
**a–e** A 4-year-old boy with Burkitt lymphoma. **a, b** Axial T2 weighted images show hypointense mass lesions that are difficult to distinguish from intestinal loops (arrows). **c, d** Diffusion-weighted images (b = 800 s/mm^2^) and (**e, f**) apparent diffusion coefficient (ADC) maps show diffusion restriction in the lesions (arrows)

GI tract lymphomas are hypoechoic and usually display circumferential wall thickening on US. The tumor generally spreads circumferentially throughout the intestinal submucosa, progressively infiltrating the bowel wall. Infiltration of the muscular layers by tumor spread leads to narrowing or dilatation in the bowel lumen whereby an aneurysmal dilatation may occur. US can also demonstrate the aneurysmal dilatation of the bowel lumen and intussusception [15] (Fig. 7a). CT shows marked focal or diffuse thickening of the intestinal wall, loss of stratification, and post-contrast soft-tissue attenuation with minimal enhancement (Figs. 7b, 8). Fat planes surrounding the tumor are preserved. Accompanying mesenteric lymphadenopathies are often seen. MRI can also depict circumferential, huge constrictive and out-growing masses with irregular bowel wall and mucosal fold thickening. Majority of the masses are homogeneous, isointense on T1-weighted images, and heterogeneous and hyperintense on T2-weighted images. After intravenous contrast material administration, the tumor shows mild to moderate enhancement [16]. Moreover, an increased signal on diffusion-weighted imaging sequence and diminished signal on apparent diffusion coefficient maps are seen (Fig. 9). PET/CT is the preferred functional imaging technique both for initial staging and to evaluate treatment response in children with Burkitt lymphoma. PET/CT has been shown to reveal disease sites that are not previously identified, possibly leading to upstaging of the disease [13].

Differential diagnosis contains inflammatory bowel disease, gastrointestinal stromal tumor, carcinoma and IMT. However, without obstruction, a bulky mass or diffuse infiltration with preservation of fat planes, multiple site involvement, and associated bulky lymphadenopathies are suggestive for lymphoma [17, 18]. Another distinctive sign is significantly lower apparent diffusion coefficient values in adenocarcinoma as compared to those of lymphoma [19].

Adjuvant chemotherapy, radiotherapy, and radioimmunotherapy are treatment modalities for the management of intestinal lymphoma.

### Leiomyosarcoma

Smooth muscle tumors are uncommon in children. Leiomyosarcoma accounts for less than 0.2% of all childhood malignancies. The most prevalent locations are (anywhere in) the GI tract. Jejunum is the most common involvement site for leiomyosarcomas in the pediatric age group. Presentation varies from none (asymptomatic abdominal mass) to occult or active gastrointestinal hemorrhage with anemia, intestinal obstruction/perforation and intussusception [20, 21]. US demonstrates different echo patterns for abdominal sarcomas including homogenously hyperechoic and hypoechoic masses as well as mixed echo pattern and cystic structures. Coarse calcification is an uncommon finding in this tumor. At CT, small tumors may be homogeneously solid, but large tumors have extensive areas of necrosis. Specific findings of these malignant tumors are central areas of lower density, surrounded by a rim of higher density peripheral areas with significant enhancement after contrast administration. MRI demonstrates a mass—which is isointense to muscle on T1-weighted images and heterogeneously hyperintense on T2-weighted MR images—containing necrosis and heterogeneous post-contrast enhancement. It is essential to keep in mind that leiomyosarcoma does not include fatty elements [22].

Surgical/en bloc resection is the most accepted treatment option. Adjuvant chemotherapy is another treatment modality in children [23].

## Gastric tumors

Most primary gastric tumors stem from epithelial cells of the stomach and are prone to be malignant in adults. In contrast children, most of the tumors (reported as isolated case reports) have mesenchymal origin and they are benign, e.g., leiomyoma, benign teratoma, hemangioma, adenoma, schwannoma, fibroma and nerve fibroma [24]. Borderline tumors of the stomach consist of IMT and epitheliomesenchymal biphasic tumor, malignant tumors of the stomach including adenocarcinoma, leiomyosarcoma, malignant germ cell tumor, rhabdomyosarcoma, lymphoma and peripheral primitive neuroectodermal tumor [25]. Primary malignant gastric neoplasms are uncommon in the pediatric age group.

### Gastric adenocarcinoma

Gastric carcinomas are also unusual in children, responsible for only 0.05% of all GI tumors [26]. Presentation, treatment, and biological behavior are similar to those seen in adults. Patients present primarily with weight loss, vomiting, abdominal pain, or a palpable abdominal mass. Patients younger than 30 years (with early disease) have better outcome, whereas those with advanced disease have worse outcomes when compared with older patients [26].

**Fig. 10:**
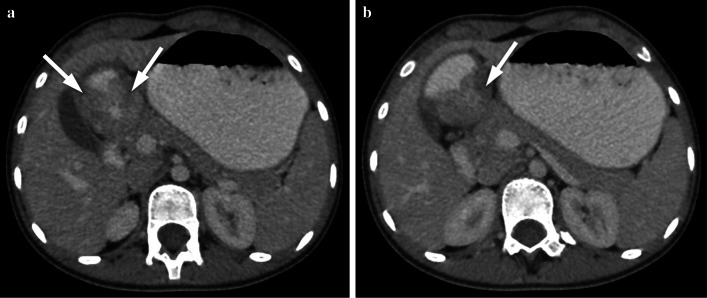
**a, b** A 12-year-old girl with gastric adenocarcinoma (signet ring cell carcinoma). She had complaints of abdominal pain, vomiting and weight loss that started 3 months ago. **a, b** Axial contrast-enhanced CT images demonstrate hypodense tumor at the antrum of the stomach (arrows) observed as pathological wall thickening

**Fig. 11:**
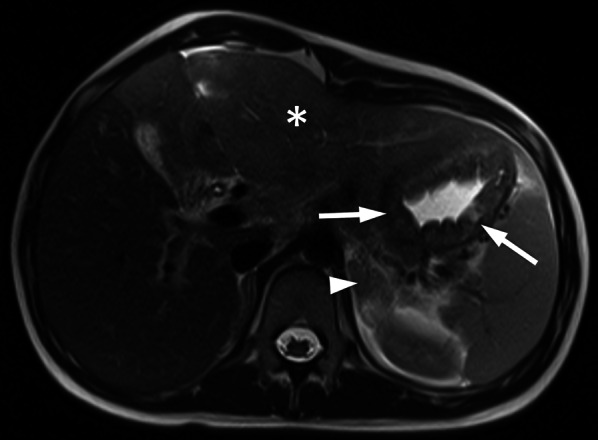
A 15-year-old girl with gastric adenocarcinoma (signet ring cell carcinoma). She had abdominal pain and anemia complaints for the last 4–5 months. Her Hb value was 6.9 gr/dL (normal range 11.7–15.5 gr/dL). Axial T2-weighted image shows diffuse increased gastric wall thickening (arrows) and diffuse hyperintense heterogeneous metastases at the left liver lobe (asterisk). Note the left adrenal mass consistent with metastasis (arrow head)

**Fig. 12:**
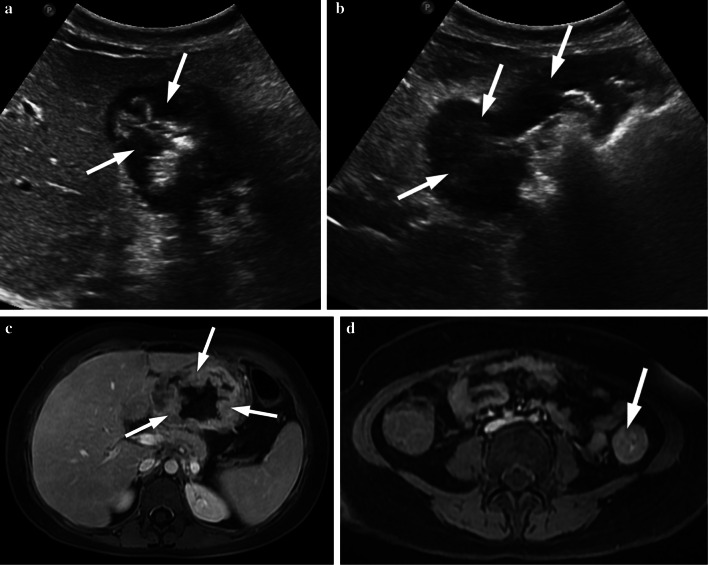
**a–d** A 16-year-old girl with gastric adenocarcinoma (signet ring cell carcinoma) and colon adenocarcinoma. **a, b** US images show increased hypoechoic gastric wall thickening (arrows). **c, d** Post-contrast T1-weighted images show diffuse increased wall thickening in the stomach and increased wall thickening in the descending colon (arrows). Postoperative histopathological diagnosis was gastric adenocarcinoma with DNA mismatch repair deficiency. The patient was diagnosed with Lynch syndrome (hereditary nonpolyposis colorectal cancer)

Gastric carcinomas are seen on US as areas of focal thickening, irregularity or distortion of the layers. US can detect the liver metastasis. CT is the most preferred technique for the evaluation of local extension of tumor, nodal disease and metastases. The particular CT finding is loss of the multilayered pattern and thickening of the gastric wall. Focal thickening greater than 5 mm on CT in a distended stomach indicates a neoplastic lesion (Fig. 10) [27]. MRI and CT findings are similar. Gastric carcinomas can appear as focal-irregular wall thickening, or as a huge circumferential mass which is hypointense on T1-weighted images and heterogeneous-hyperintense on T2-weighted images (Fig. 11). After intravenous contrast material injection, the lesion has heterogeneous enhancement and surrounding fat stranding [28]. Lower ADC values and diffusion restriction have been shown to be associated with higher TNM classification [29, 30] (Fig. 12).

The widest spread types of metastasis in children with gastric malignant tumors are lymph node metastasis and direct invasion to adjacent tissues [31]. The fundamental treatment for gastric tumors is surgical resection. For sure, a metastatic workup is vital to specify the type of surgical resection.

### Gastrointestinal stromal tumors

Gastrointestinal stromal tumors (GIST) are the most prevalent mesenchymal tumors of the GI tract [32]. They occur in the stomach (50–60%), small intestine (30–35%), colon and rectum (approximately 5%) and less commonly in the esophagus [32, 33]. The frequent finding in childhood GIST is anemia (86.4%) which manifests as acute or subacute bleeding due to the mucosal ulceration [33]. Tumor behavior and risk of malignancy rely on size (tumor size is < 5 cm), low mitotic index and absence of lymphadenopathy [34–36]. GISTs smaller than 5 cm are generally regarded benign with a very low risk of recurrence. GISTs have unique immunohistochemical profile that is useful for confirming the diagnosis. In 95% of the patients, it is significant to determine *KIT* (CD117) which is a tyrosine kinase receptor in the interstitial cells of Cajal [35].

**Fig. 13:**
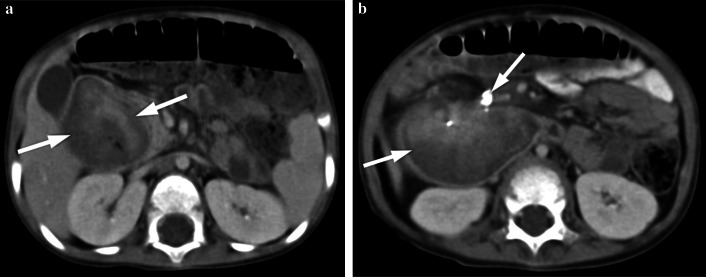
**a, b** A 3-year-old boy with duodenal gastrointestinal stromal tumor who had been complaining of vomiting for 3 months. The mass was palpable in physical examination. **a, b** Contrast-enhanced axial CT images show duodenal tumor with calcifications (arrows)

**Fig. 14:**
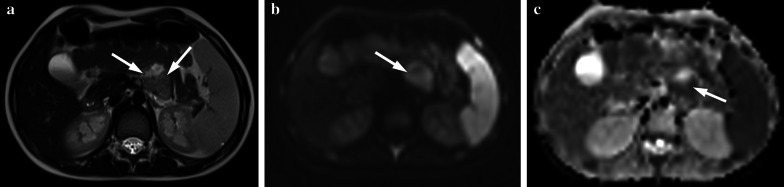
**a, c** A 9-year-old girl was admitted to the pediatric emergency department with complaints of black stool and fatigue. She had several admissions to various gastroenterology departments during the last 2 years due to recurrent melena and fatigue. In the laboratory tests; Hb was 7.8 gr/dL (normal range 11.7–15.5 gr/dL) and ferritin was 8.3 ng/mL (normal range 20–336 ng/mL). Meckel’s diverticulum scintigraphy was normal. Esophagogastroduodenoscopy and ileocolonoscopy showed a normal appearing mucosa. Abdominal US was normal. **a** Axial T2-weighted MRI shows heterogeneous mass with intratumoral cystic portion at proximal jejunum (arrows). **b** Diffusion-weighted image (b = 800 s/mm^2^) and (**c**) apparent diffusion coefficient (ADC) map show diffusion restriction of solid portion of the tumor (arrows). Segmental resection was performed and the histopathological diagnosis was jejunal gastrointestinal stromal tumor

Radiological findings of GISTs vary, based on the size and aggressiveness of the tumor. Large GISTs are, eccentric, well-circumscribed masses on CT and/or MRI and they are often heterogeneous due to necrosis, hemorrhage, or cystic degeneration during the time of presentation. Cavitation, ulceration and fistulation to the gastrointestinal lumen can be detected in GIST. After contrast material administration, these tumors are hypervascular with heterogeneous enhancement on CT and with intense arterial enhancement on MRI (Fig. 13). Small tumors are commonly depicted as submucosal or endoluminal polypoid round-shaped masses with homogenous contrast enhancement [36–38]. A low ADC value and diffusion restriction are suggestive for high malignancy risk [37] (Fig. 14).

Gastric leiomyoma, schwannoma, and leiomyosarcoma should be considered in the differential diagnosis. However, GIST tumors are hypervascular on early arterial phase and hypoenhancing as compared to the surrounding tissues on portal venous phase [38].

Surgery (en bloc resection) is the most accepted treatment.

### Teratoma

Teratomas are embryonal tumors derived from pluripotent cells. They usually consist of elements from all the three germ layers, i.e., ectoderm, endoderm and mesoderm. In the GI tract, they may be located in the stomach, pancreas, mesentery, omentum, etc. Gastric teratomas are very rare, and they are responsible for less than 1% of all teratomas seen in neonates and infants. Histologically, teratomas are classified under three subgroups: mature, immature and malignant form. Although rare, malignant gastric teratomas have been reported in the literature. These tumors usually present with abdominal mass, abdominal pain, obstruction, or hematemesis [39–41]. US demonstrates a quite large, well-marginated, heterogeneous mass including cystic or solid components with smooth borders, but might also display fat and calcification. On CT scans, a well-defined, mixed cystic and solid mass with calcifications and fat density is seen. On MRI, the sebaceous component of the mass is hyperintense on T1- and T2-weighted images [41]. Diffusion-weighted imaging displays diffusion restriction within the mass [41]. Fat and coarse calcification content help to exclude other tumors in children. The treatment of gastric teratoma is complete/wide resection.

## Esophageal tumors

Esophageal tumors are scarcely seen in the pediatric age group—limited to anecdotal case reports in the literature. Reasons behind the onset of esophageal tumors in younger age groups are uncertain. Esophageal epithelial tumors occur due to chronic irritation from a wide range of known environmental carcinogens and gastric contents in chronic reflux. Theilen et al. reported that approximately 31% patients with esophageal tumors were diagnosed with Barret esophagus and 63% had preexisting conditions such as spinal palsy, hiatus hernia, esophageal atresia, which were associated with gastroesophageal reflux disease as a risk factor for Barrett’s esophagus [2]. Dysphagia with significant weight loss and presence of a mass in the distal esophagus are the most frequent findings at the initial diagnosis of esophageal cancers.

Contrast barium studies can be utilized to examine the esophagus and esophagogastric junction, and they are considered as sensitive techniques for detecting the carcinoma except for early stage. Benign or malignant strictures that are significant for the tumor should be correlated with endoscopy. Moreover, early-stage esophageal cancers may not be detected in barium studies. Hence, endoscopic evaluation of any suspected abnormality should be performed.

**Fig. 15:**
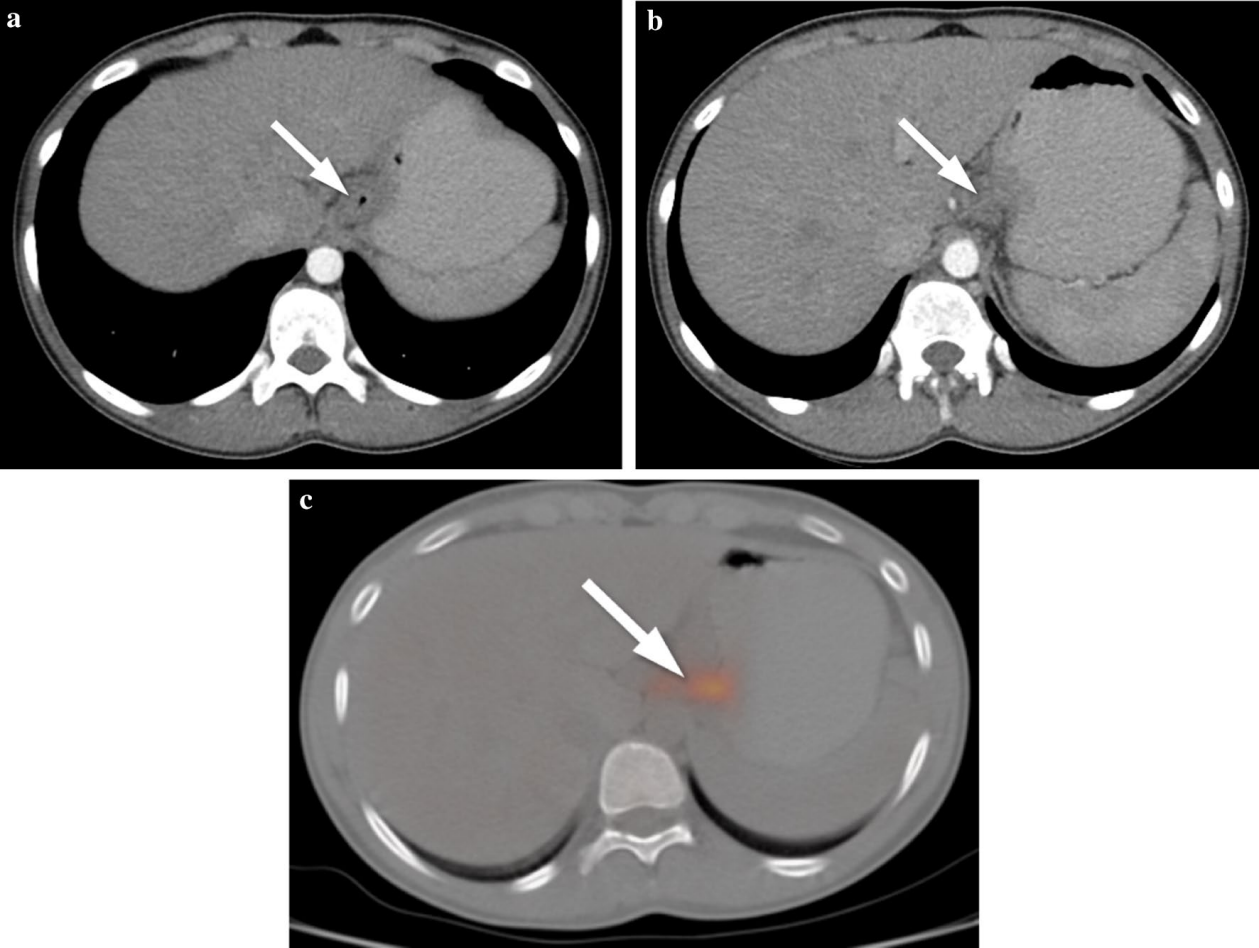
**a**–**c** A 14-year-old girl with esophagus adenocarcinoma. **a, b** Axial contrast-enhanced CT images show diffuse hypodense wall thickening at the distal part of the esophagus (arrows). **c** PET/CT image shows focal hypermetabolic activity at the distal part of the esophagus corresponding to the mass seen on CT

The most important aim of CT and MRI in esophageal cancer is to ascertain the stage of the disease as well as to plan for the treatment [42–45]. Normal diameter of the esophageal wall is 3 mm on CT when it is distended. A wall thickness of > 5 mm is specified abnormal [45]. Asymmetric thickening of the esophageal wall is a principal but nonspecific CT finding in esophageal cancer. The disappearance of fat planes between the tumor and adjacent structures in the mediastinum, and displacement/indentation of other mediastinal structures are accepted as local invasion on CT. Additionally, CT may help to screen enlarged lymph nodes in the mediastinum and celiac regions, tracheobronchial fistula or tumor extension into the airway lumen [46] (Fig. 15). Although MRI findings of esophageal carcinomas are limited in the literature, these tumors appear as areas of iso-intensity relative to the residual esophageal wall on T1-weighted images and as intermediate-signal-intensity to high-signal-intensity on T2-weighted images. Metastatic lesions in the liver are seen as iso-hypointense foci relative to the normal liver on T1-weighted images and as hyperintense foci on T2-weighted images [46]. The differential diagnosis based on imaging findings includes esophageal leiomyoma and leiomyomatosis. Herein, stranding in the periesophageal soft/adipose tissues and the presence of local invasion or tracheobronchial fistula are significant for malignancy. Since esophageal tumors are very rarely observed in children, treatment is based on principles used in adults: surgery, radiotherapy, and chemotherapy are several treatment options.

## Conclusion

Gastrointestinal (GI) tract tumors are rarely seen in children and have a different and broad histopathological spectrum. Awareness and early diagnosis are critical. Different tumors have different findings. Besides, the most frequent tumor in children is ileocecal or ileum lymphoma, and they rarely manifest as necrotic or heterogeneous mass. Not only gastric/colonic but all GI tumors present with increased wall thickening. We strongly imply that—despite a normal US evaluation—CT and MRI should be performed in clinically suspected cases for prompt diagnosis/management.

## Data Availability

The datasets used and/or analyzed during the current study are available from the corresponding author on reasonable request.

## Abbreviations

CT: Computed tomography
GI: Gastrointestinal
GIST: Gastrointestinal stromal tumor
IMT: Inflammatory myofibroblastic tumor
MRI: Magnetic resonance imaging
NHL: Non-Hodgkin’s lymphoma
NSAID: Non-steroidal anti-inflammatory drug
US: Ultrasonography
